# Factors that enable effective One Health collaborations - A scoping review of the literature

**DOI:** 10.1371/journal.pone.0224660

**Published:** 2019-12-04

**Authors:** Kaylee Myhre Errecaborde, Katelyn Wuebbolt Macy, Amy Pekol, Sol Perez, Mary Katherine O’Brien, Ian Allen, Francesca Contadini, Julia Yeri Lee, Elizabeth Mumford, Jeff B. Bender, Katharine Pelican

**Affiliations:** 1 Veterinary Population Medicine Department, One Health Division, College of Veterinary Medicine, University of Minnesota, St. Paul, Minnesota, United States of America; 2 Veterinary Population Medicine Department, Center for Animal Health and Food Safety, College of Veterinary Medicine, University of Minnesota, St. Paul, Minnesota, United States of America; 3 Department of Veterinary Epidemiology and Public Health, School of Veterinary Medicine, University of Surrey, Guildford, United Kingdom; 4 City of Minneapolis Health Department, Food, Lodging and Pools, Minneapolis, Minnesota, United States of America; 5 One Health Country Operations Team, Department of Country Health Emergency Preparedness and IHR, World Health Organization, Geneva, Switzerland; 6 Environmental Health Sciences, School of Public Health, University of Minnesota, Minneapolis, Minnesota, United States of America; San Diego State University, UNITED STATES

## Abstract

Advocates for a One Health approach recognize that global health challenges require multidisciplinary collaborative efforts. While past publications have looked at interdisciplinary competency training for collaboration, few have identified the factors and conditions that enable operational One Health. Through a scoping review of the literature, a multidisciplinary team of researchers analyzed peer-reviewed publications describing multisectoral collaborations around infectious disease-related health events. The review identified 12 factors that support successful One Health collaborations and a coordinated response to health events across three levels: two *individual factors* (education & training and prior experience & existing relationships), four *organizational factors* (organizational structures, culture, human resources and, communication), and six *network factors* (network structures, relationships, leadership, management, available & accessible resources, political environment). The researchers also identified the stage of collaboration during which these factors were most critical, further organizing into *starting condition* or *process-based* factors. The research found that publications on multisectoral collaboration for health events do not uniformly report on successes or challenges of collaboration and rarely identify outputs or outcomes of the collaborative process. This paper proposes a common language and framework to enable more uniform reporting, implementation, and evaluation of future One Health collaborations.

## Introduction

Ongoing and emerging health challenges such as infectious disease epidemics, bioterrorism, antimicrobial resistance, and natural disasters require a coordinated response from a highly diverse, collaborative, and trained health workforce. “One Health” is a concept and approach intended to meet such demands. Though loosely defined, a broadly accepted definition of One Health describes it as “*the integrative effort of multiple disciplines working to attain optimal health for people*, *animals*, *and the environment* [1–4] World Organisation of Animal Health (OIE), n.d.; World Health Organization, n.d). A One Health approach recognizes that complex health challenges are beyond the purview of any one sector or discipline working in isolation [5] and that a resilient health workforce must be capable of effective and collaborative prevention and detection of, as well as response to emerging health challenges. A One Health approach, therefore, calls for collaboration across disciplines, sectors, organizations, and national borders in support of increasingly complex health challenges [1–2,4–8].

While One Health advocates increasingly support collaborative and multi-sectoral approaches to health challenges, no common language or metrics exist to uniformly describe and evaluate such efforts. Few studies explicitly analyze the factors and conditions that support effective One Health practices and collaborations. This hinders the ability of health professionals to learn from past experiences and improve upon current and future One Health policies, partnerships, and practices. This paper seeks to address this gap by analyzing and identifying factors that enable effective multisectoral collaboration and response to health events.

In this study, a multidisciplinary team of researchers reviewed a broad scope of literature describing collaborative and multi-sectoral approaches to past health events to understand how such collaborations are commonly described and evaluated and to identify and synthesize enabling factors for One Health collaborations. This paper identifies twelve factors related to effective One Health implementation and collaboration and concludes with a proposed framework for evaluating future multisectoral One Health collaborations. The ultimate aim of this work is to support and improve multisectoral preparedness and response efforts.

### Background on One Health

Although its conceptual foundations date back hundreds of years, the formal global health construct known today as One Health wasn’t officially recognized by international and scholarly bodies until 1984 [8]. The HIV/AIDS pandemic in the 1980s and the Hanta virus outbreak in 1993, made clear that emerging disease threats can cross national borders, cultures and species. With that came a broader recognition that animal and zoonotic diseases pose a serious threat not only to human health but to global health security. Policy makers and health practitioners looked to collaborative health efforts as a response to these emerging challenges [3].

The subsequent decades which were marked by unprecedented global interconnectedness and human mobility [9] were associated with threats to global health security, including man-made threats, such as the use of anthrax as a bioweapon, and emerging diseases like SARS and avian influenza. These challenges necessitated the need for a more formal coordinated action from countries, regions, and the global health community at large to address such health threats.

In order to address the afore-mentioned challenges, there have been emergence of major health initiatives and frameworks such as the Global Health Security Agenda, the International Health Regulations-Joint External Evaluations (IHR-JEE), the World Health Organization (WHO)-World Organisation for Animal Health (OIE) Operational Framework for Good Governance at the Human-Animal Interface [10], and the World Bank’s One Health Operational Framework [11]. A common thread among these initiatives is the emphasis on multisectoral and transdisciplinary collaboration and a call for strengthening human, animal and environmental health systems through a One Health approach.

The global health community, including those already engaged in One Health, continue to grapple with the fundamental questions of what characterizes a successful One Health approach, including how to set goals, establish frameworks, facilitate collaborative work, and how to process and measure outcomes [12]. Efforts to measure One Health programmatic outcomes and operations are necessary for the improvement of collaborative efforts. This article supports such efforts by 1) identifying key factors that support effective collaboration around health events and 2) proposing a framework for documenting and evaluating One Health collaborations in a more uniform and systematic manner.

### Conceptual framework: Understanding One Health collaborations

Collaboration is an inherent and explicit part of the One Health approach which calls for the active engagement of institutions, managers, and health practitioners across disciplines and sectors [1–4]. Despite widespread recognition of the importance of a One Health approach, there exists a gap in the literature regarding what constitutes a successful One Health collaboration. This study draws upon the existing public affairs literature on collaborative, or ‘cross-boundary’ collaborations to understand which factors enable successful collaboration around health events.

#### Review of the literature on collaboration

Scholars of public policy, organizational partnerships, team science, and multisectoral collaboration have produced a series of theoretical frameworks to describe cross-boundary collaborations and identify which practices make them successful [13–15]. The focus on collaboration and partnership is not unique to any one discipline, yet there is very little cross-fertilization of research across disciplines. This research builds upon the existing literature on cross-boundary collaborations and applies it to One Health collaborations. The conceptual framework for this study focuses on three critical phases of a successful cross-boundary collaboration: adequate *starting conditions*, an effective *process* of collaboration, and attention to the outcomes of collaboration [16–20].

#### Starting conditions

There is a general consensus in the literature on cross-boundary collaborations that *starting conditions-*the conditions in place before any collaborative process begins—impact the process, structures and outcomes of collaborative engagement. These include prior history (e.g. successes, failures, existing partnerships), the environment (e.g. resource imbalances, stakeholder incentives), and relational dynamics (e.g. balances of power, who convenes or facilitates the collaboration, and how and by whom problems are defined) [16,17]. The presence or absence of such conditions influences successes and challenges encountered during the collaborative process.

#### Process

Beyond starting conditions, many scholars point to the *process* of collaboration itself and the structures in place to support effective collaboration [13,14,20,21]. Although the terms used for collaboration vary, scholars focusing on the process of collaboration point to the importance of leadership, shared goals, trust and mutual understanding, institutional structures and resources, communication, and data management.

#### Measuring outcomes

A review of the literature on collaboration suggests a lack of validated metrics for measuring collaborative effectiveness and performance. Several scholars of cross-boundary collaborations, citing works published between 2005 and 2019, highlight the importance of measuring the outcomes of collaboration and lament the challenges of describing and evaluating collaborations in a uniform way [12,14,16,17,20,22–24]. This underscores the importance of understanding which factors support collaborative efforts, and how teams can evaluate their performance and outcomes in association with these factors.

The literature on cross-boundary collaborations and its attention to the starting conditions, processes, and outcomes of collaborative approaches have informed this study on the factors that enable effective One Health collaborations. The following questions guided this study: What factors (systems, structures, processes, skills, competencies, decisions, and actions) enabled two or more disciplines or sectors to collaborate effectively in a health event?

## Methods

### Scoping review

A scoping literature review was conducted to identify key factors that facilitate multisectoral collaborations around major health events such as disease outbreaks using published accounts of actual health events. A scoping review, in contrast to a systematic review, is well-suited for a field such as One Health that is still relatively new and evolving, as the method allows for assessment of emerging evidence, as well as a first step in research development [25][p. 12]. Due to the lack of a common language and framework for describing One Health collaborations, this scoping review builds that foundation by providing a broad overview of One Health collaborations and supporting the synthesis of key concepts, evidence, and research gaps [26,27].

The scoping review was initiated by a multidisciplinary team in January 2017. The team members were composed of individuals with expertise in veterinary medicine, public health, public policy, organization and management leadership studies, international development, monitoring and evaluation, and education. Because the researcher is central to the methods and analysis of qualitative research, it was important to select a transdisciplinary research team that could work effectively to address the research questions and to illustrate the disciplines that were represented in the transdisciplinary approach employed for this scoped review.

#### Selection of relevant articles

The search included peer-reviewed articles available to-date in the U.S. National Library of Medicine’s PubMed database that were searched using specified MeSH (Medical Subject Headings) terms. Although the multidisciplinary research team has extensive experience in One Health, they were not trained in sensitive search strategies [26]. The research team thus elected to work with a University of Minnesota research librarian to develop MeSH terms for this study. Table 1 provides a list of the key terms used to identify articles discussing multisectoral health events and collaborations. To avoid tautology, it was a deliberate decision to not use “One Health” as a search term. Instead, drawing upon the researchers’ extensive experience in One Health, various terms were used to describe One Health and similar multidisciplinary and cross-sectoral health collaborations. The underlying assumption was that any articles explicitly addressing One Health would be captured using these key terms. This initial MeSH search identified 2,630 non-duplicated articles. This scoping review was an inductive study of the literature and was conducted in order to support more hypothesis driven research for One Health. By design, the authors elected to limit this literature review to the PubMed database at the outset of the study. PubMed is peer-reviewed and peer-led database. Articles are selected and included based on scholarly and quality criteria by literature review committees and are tagged by keyword and by article structure, contributing to more accurate retrieval than other databases (e.g. Google Scholar); accurate retrieval supports the search results are reproducible and reportable, which is critically important for a scoping review of the literature in which it is important for other researchers, no matter their location, to repeat the study. The decision to use one database reflects the exploratory nature of this study and the Author’s intent to propose further hypothesis-driven research that may include additional databases. This methodological choice is in line with Arksey and O'Malley (2005) who attest that decisions must be made at the outset of a study to clarify reasons for the coverage of the review in terms of time span and language [26] [p.23-24].

**Table 1:** Article search terms (yielded 2,630 unduplicated results).

MeSH Terms Searched	Key Terms (All Fields) Searched
Communicable Disease Control Population Surveillance Zoonoses/epidemiology Zoonoses/organization & administration Zoonoses/prevention and control Disease Outbreaks/epidemiology Disease Outbreaks/legislation & jurisprudence Disease Outbreaks/organization & administration Disease Outbreaks/prevention and control Interprofessional Relations Cooperative Behavior Community Networks/organization & administration	Multidisciplinary/Multidisciplinary/Multidisciplinary Trans-disciplinary/Trans disciplinary Cross-sectoral/Cross sectoral
Community Networks/legislation & jurisprudence International Cooperation/legislation & jurisprudence International Cooperation/manpower International Cooperation/organization & administration Efficiency, Organizational/organization & administration Organizational Innovation/legislation & jurisprudence Organizational Innovation/methods Organizational Innovation/organization & administration Organizational Culture	

**Fig 1:**
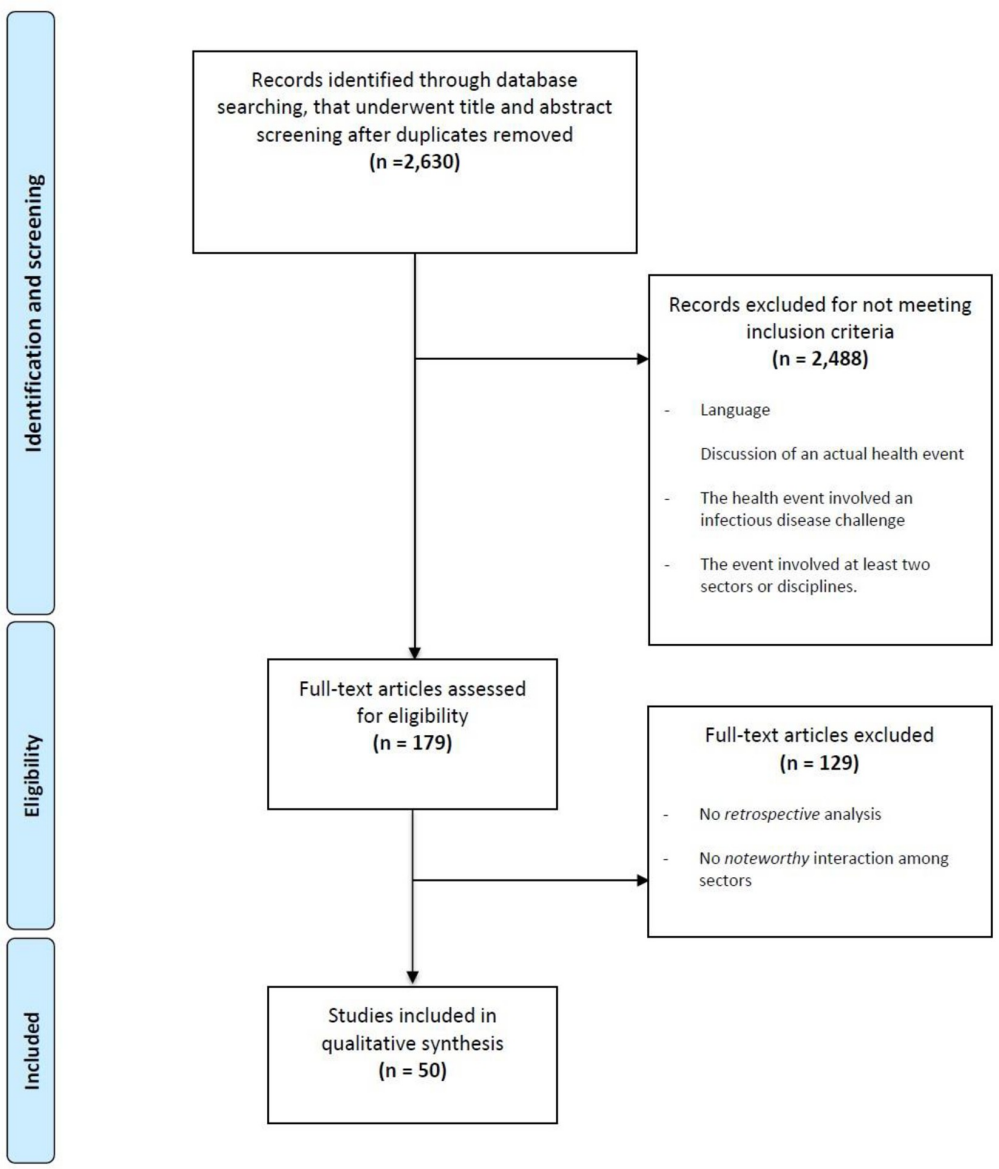
PRISMA diagram.

Initially, citations and abstracts of these articles were screened in two phases. The articles were reviewed for inclusion based on the criteria outlined in Table 2. In the first screening, 179 abstracts met initial inclusion criteria and full articles were procured and reviewed. In the second phase of screening, two further criteria were included to better achieve scoping review objectives. The research team divided into transdisciplinary pairs which included a reviewer from the health sciences and one from the social sciences. Each of the articles that met the initial inclusion criteria were divided among the team members and then independently reviewed according to the modified screening criteria. Articles were included if both reviewers agreed that they met all initial requirements. In instances where the transdisciplinary reviewers did not agree, the articles were brought to a full research team meeting and reviewed jointly until consensus among all researchers was achieved. This same method of collaborative review was used for the second round of screening and resulted in 50 articles for the final analysis. The PRISMA diagram below (Fig 1) illustrates the article search, screening, and review process.

**Table 2:** Article screening criteria.

Initial Screening Criteria (yielded 179 results)	Modified Screening Criteria (yielded 50 results)
Inclusion Criteria: 1. Full articles written in English, Spanish, French, Portuguese, or Italian; 2. Article discussed an actual health event; 3. The health event discussed involved an infectious disease challenge; and 4. The case or event involved at least two sectors or disciplines.	Inclusion Criteria: 1. Articles met initial screening criteria and were included if they met the following targeted requirements: 2. The article provides a *retrospective analysis* of an actual health event; 3. The case or health event involved a *noteworthy* (describing successes or challenges encountered during health event) interaction among at least two sectors or disciplines. Exclusion Criteria: Articles were excluded if they failed to discuss any specific aspects of collaboration, even if they generally acknowledged the importance of multisectoral collaboration.

#### Analysis

The interdisciplinary team conducted an analysis of the 50 articles that explicitly addressed multisectoral collaboration in response to an actual health event. Each reviewer coded approximately 5–10 selected articles using the qualitative data analysis software, MaxQDA [28]. Descriptive codes were identified in advance to ensure that baseline data reflected the One Health aspects of the articles reviewed. All other codes emerged from the data using a grounded theory approach [29,30]. Preliminary and axial coding procedures are outlined in the following section and ensured that inductive and deductive thinking could be related.

#### Preliminary coding

A set of predetermined, descriptive codes were used to denote the location and nature of the health event in the articles, including specific infectious agents, relevant disease vectors or hosts, and the various entities involved in the collaboration. Each paper was coded for the predetermined codes outlined in Table 3.

**Table 3:** Preliminary descriptive codes.

1. Type of health event reported in the articles 2. Location of health event 3. Infectious agent 4. Entities involved in the response (roles, disciplines, sectors)

Predetermined codes were also used to identify the entities involved in each health event response. The team used the code “roles” to identify individuals or groups who participated in the coordinated response in a formal role based on individual expertise and formal training. While many of these roles represent professions in the health sciences, this category also included representation from the social sciences, media and community relations, government, and engineering. Other articles focused on types of training, identified by the research team as “disciplines,” (e.g., *clinical epidemiology* [31] or *food safety* [32]), or specific professions (e.g., *toxicologist* [33] or *information technology specialist* [34] versus specific professions). A third type of classification in the literature was more general categorization of sectors involved, such as the traditional designations of *Public*, *Non-profit*, *and Private/For-profit*.

#### Axial coding

Axial coding was used to construct linkages between “data sets” or, in this scoping review, articles regarding intersectoral collaboration. Axial coding is a qualitative research technique to relate data together in order to reveal codes, categories, and subcategories, as well as patterns in the data [35]. This grounded theory is an iterative process that combines inductive and deductive thinking.

Each article was first coded to identify any area of text where authors analyzed collaboration around a specified health event. In this process, it became quickly apparent that the review team would need to differentiate between actual and hypothetical forms of collaboration reported. All articles included in the analysis at this stage were retrospective analyses of actual health events, yet many were actually prospective in their analysis and discussion. As an example, several of these articles included suggestions based on what should happen in an ideal scenario, rather than what occurred in practice, thus leaving out key details of the actual event. Therefore, a first round of organizing codes differentiated between collaborations that actually happened versus ideal scenarios and hypothetical lessons, allowing the research team to focus the analysis on what actually happened (Table 4). The text was further coded to reflect whether the authors were reporting a success factor of collaboration, or a challenge of collaboration. Both the successes and challenges reported in the literature were related during the grounded theory thematic analysis and informed the final thematic results reported.

**Table 4:** Organizing codes.

Code 1: Collaborative analysis–Success reported/ Actually happened during reported health event Code 2: Collaborative analysis–Challenge reported/ Reflection on what should happen during future health events

After the first round of axial coding was conducted to organize the data, the authors employed a deductive framework developed from the review of literature on multisectoral collaboration [13]. Aligned with this framework, the research team distinguished between starting conditions for collaboration, the process of collaboration itself, and the outcomes of collaboration (Table 5).

**Table 5:** Deductive codes informed by the literature.

Code 1: Starting conditions of collaboration Code 2: Process-based conditions of collaboration Code 3: Outcomes of Collaboration

Finally, the review team re-examined the passages coded as “actually happened” and “successes”. These codes were then related to the deductive codes of starting conditions and process-based factors. An Excel table was used to organize axial codes into a table of final results.

#### Limitations

The primary limitations of this scoping review are three-fold. First, the literature analysis relies on peer reviewed publications alone, which may have underrepresented collaborative efforts that are more commonly encountered in grey literature. Future work may be expanded to include these types of sources.

Second, there was no consistent framework or language for reporting the successes or challenges of collaboration, and thus, important content may have been missed during the search and review [36]. The scoping review team tried to overcome this with two strategies, which included building an expanded list of search terms and conducting an iterative review process using two independent transdisciplinary reviewers. Both methods offset this limitation and might have minimized the likelihood of missing specific content. Third, the researchers could not identify specific metrics for evaluating performance and collaboration in the literature. This meant that an evaluation baseline was not present. However, the research team believes that the final subset of articles represents a diverse crosssection of transdisciplinary efforts around emerging health events.

## Results

The scoping review yielded 50 peer-reviewed publications explicitly addressing multisectoral collaboration in response to an actual health event. This section describes the nature of the One Health collaborations analyzed as well as the various factors that enable One Health collaboration.

### Descriptive results

Types of One Health events analyzed include natural disasters, infectious disease outbreaks, endemic disease, bioterrorism, and biosecurity preparedness. In each of these cases, the underlying multisectoral collaboration was either a preparedness (planned or ongoing collaboration) or response (emergency or ad hoc collaboration) effort. The sample included One Health events from around the world. Most articles addressed health events in Europe/Eurasia (25%), the Americas (25%), and Asia (23%). Less represented in this sample were health events taking place in Africa (11%), Oceania (10%), and the Middle East (6%).

Most health events involved a specific infectious agent (97%), while the remaining 3% focused on infectious disease challenges such as hospital infections, pest management, or tsunami response. A total of 67 different infectious agents were coded. Among the infectious agents identified, 58% were bacterial, 40% were viral, and 2% were protozoal (e.g. malaria). 39% of these agents primarily affect humans and 33% are predominantly animal-related. 16% of the agents were food and water-related, 10% were insect related, and an additional 2% were related to environment. Overall, 60% of the infectious agents were considered zoonotic, meaning they spread between humans and animals. Relevant disease vectors or hosts represented in more than one publication included bats, cattle, poultry, horses, swine, humans, mosquitoes and midges.

Involved parties or entities played varied roles and represented diverse disciplines and sectors, as illustrated in Table 6.

**Table 6:** Codes for “entities” involved in the multisectoral event.

Roles (in order of code frequency)	Disciplines (in order of code density)	Sectors (in order of code density)
• Medicine • Health Sciences General/Not defined • Government • Animal Health • Technical Support • Environmental Health • Social Sciences • Media	• Human health • Animal health • Environmental health	• Public sector • Non-profit sector • Private/for-profit sector • Academic Institutions

### Thematic results

Thematic findings are presented according to the One Health collaboration framework, which distinguishes between individual, organizational, and network factors that enable multisectoral and transdisciplinary collaboration at the onset and in the process of addressing a One Health event. The team ultimately created organizing categories that reflected the *individual*, *organizational* and *network* levels of collaboration (Table 7). These categories were informed by a review of the literature; for the purposes of this discussion, the definition of network is provided by Emerson and Nabatchi [18], and is defined as “the processes and structure of public policy decision making and management that engage people constructively across the boundaries of public agencies [organizations], levels of government, and or the private and civic spheres in order to carry out a public purpose that could not otherwise be accomplished,” [p.2]. Within each level, the review team created groups of subcategories to further organize codes.

**Table 7:** Emergent axial codes organized into levels of the collaborative network.

**Individual collaborative factor themes:** Education and Training Prior Experience & Existing Relationships Ad hoc “just-in-time” training
**Organizational collaborative factor themes**: Organizational Structures: Policies and Protocols, Systems Organizational Culture: Leadership, accountability, ownership, trust, transparency of processes, existing relationships, systems based thinking, cultural awareness and engagement Human Resources: Prior Experience & Relationships, Staffing/ Roles & Responsibilities, Reflexive workforce
**Network collaborative factor themes:** Network Structures: Structures & Coordinating Mechanisms, Established Roles & Responsibilities Network Relationships: Preemptive Planning, Relationships & Partnerships, Diverse/Inclusive Stakeholder Engagement Existing Resources: Human Resources/Skilled Professionals, Financial Resources/Funding
Political environment Network Leadership Network Management: Task Management, Communication, Awareness, Ongoing Stakeholder Engagement Monitoring and Evaluation (M&E) Resource mobilization & allocation: Material distribution, Human Resource mobilization

The research team identified 12 key factors that support successful multi-sectoral collaborations around major health events. At the individual level, these factors include 1) education & training and 2) prior experience & existing relationship. Organizational factors include 3) organizational structures, 4) organizational culture, 5) human resources, and 6) communication. Finally, network-level factors include 7) network structures, 8) relationships, 9) leadership, 10) management, 11) available & accessible resources, and 12) the political environment. These final individual, organizational and network factors were then further characterized according to their relevance at the start of a collaboration “starting condition” or during the process “process-based” factors of collaboration. The researchers identified that the organizational thematic factors were relevant to both starting conditions and process-based factors so were not separated. The final results of this literature review are thus presented in Table 8.

**Table 8:** Final axial coding process included both inductive and deductive codes and reflects emerging themes for successful collaboration.

Levels	Starting Condition Factors Initial deductive code (Table 5)	Process Factors Initial deductive code (Table 5)
***Individual*** (Emergent Axial code Table 7)	***Individual Factors*** **Education and Training (including skills & competencies)** Preemptive technical training/ continuing education [37–45] Disease specific technical training [34,45,46] Preemptive collaborative training [47,48] Strong public-sector led training [39] training and capacity building provided a platform for better collaboration for outbreak [49] NGOs support gov. through staff training [50] Participatory epidemiology training [51] **Prior Experience & Existing Relationships** **(informal/formal)** Pre-existing multisectoral relationships [45,52–55] Previous experience collaborating for health events [34,56,57]	***Individual Factors*** **Ad hoc “just-in-time” training** Shared training & organizational alignment; aggressive, rigorous, just-in-time, and critical trainings for key positions and critical events with monthly follow-up meetings to support compliance [31,58] Training and capacity building provided a platform for better collaboration for outbreak response [49] Instituting multisectoral disease specific training; Ongoing training—for new and existing systems [39,45]
***Organizational*** (Emergent Axial code Table 7)	***Organizational Factors (applicable to both starting conditions and process of collaboration)*: Structures** **Policies and Protocols** Shared response guidelines [42,50] Structures frequently included policies/protocols [59,60] Reporting -Management protocol -Task Management -Response Plan -Communications/ communication strategy [34,61] Infection planning, control and traceback procedures [62] **Systems** Reporting, laboratory systems [59] Surveillance systems [41,58,59] Planning; Iterative Improvement of systems [46,48,60] Information management system/ database [41,48,63,64] Information Sharing (data available and useful) [45,48]) Tool sharing during response [65] Lab systems in place [59] Online system for HR recruitment [45] Intentional multidisciplinary engagement, collaborative capacity [43,48,66,67] Standard operating procedures [55] Interoperability [42] Needs assessment and prioritization [38,48] **Culture** ***Leadership*, *accountability*, *ownership*, *trust*, *transparency of processes*, *systems based thinking*, *cultural awareness and engagement*** Leadership to support the iterative and developmental review of collaborative processes [58] Strong, engaged Leadership [32,35,52,68] Accountability; Ownership [67,68] Cultural Engagement; Engagement; Diversity/ Involvement of community [67,69] Trust [38,41,49,70] Transparency [31,34,61] Need to understand each other’s' processes [53,70] Systems based thinking/ approach [34,48] Cultural awareness; engagement of diverse stakeholders to reflect community needs [53] Credibility [38] **Human Resources** ***Prior Experience & Relationships*** Existing Relationships [49] Institutional Knowledge (experience and relationships) [31,45] Revise and revisit mandates based on lessons learned [37,71] ***Staffing/ Roles & Responsibilities*** Clearly defined roles and responsibilities [35,42,65] Resources available and accessible (including Human Resource allocation) [35,45] Informed staff/ staff are aware of systems in place, increased engagement of staff [31,45] ***Reflexive workforce*** Reflexive Human Resource Protocol to ensure positions are adequately filled & workers are incentivized [31,57] Reflexive approach [31,45] Adaptability to rapidly changing context [42] Rapid start-up response; shared response guidelines [42]	
***Network*** ***(in/formal)*** (Emergent Axial code Table 7)	***Network Factors*** **Network Structures** ***Structures & Coordinating Mechanisms*** Multi Sectoral Coordinating Mechanisms/ platforms for engagement [34,41,45,52,60] Memoranda of Understanding, Terms of Reference or bilateral agreements to support the development of existing relationships that promote ongoing engagement [41,45,48,72] Use of the Incident command system (ICS) [60] Creating shared protocols—platform for scientific engagement, information/ tool sharing during response [65]Reporting structure [49,60] Creating shared protocols [45] Policies -Institutional—nation-nation/ regional agreements [45,49,58,72]Basic public health and infection control measures including contact tracing, infection control procedures, and quarantine [62] Joint tasks forces and bilateral agreements ie. the crossborder task force and bilateral agreement between public hospitals [42,48,72] Jointly developed procedures to ensure coordinated investigation and cross-sector data exchange [72] Presence of Lead agency/ Task Force [41] Establish committees/ subcommittees [48,73] ***Established Roles & Responsibilities*** Clearly defined and previously established roles and responsibilities [34,42,65,72] Establish a framework with clearly established partnership roles and responsibilities [42] Identification of an inter-agency/ interdisciplinary liaison [31,73] **Network Relationships** ***Preemptive Planning*** Preemptive planning for potential disease threats (ex: MERS CoV, SARS, Ebola, etc.) [45] Creating common goals across the network [47] Setting goals [34] Local preparedness and logistics [43] ***Relationships & Partnerships*** Established/ preexisting relationships & partnerships [45,55,74] Established forum for information sharing, developing relationships, building capacity [49] Partnerships with clearly defined roles and responsibilities [40,42,49,54] Partnerships include public-private partnerships [49], NGO and donor partnerships [42], training and capacity building partnerships [40] Partnership with community centers that work with vulnerable populations [59,75] Partnership with external/ global organizations to support response [62,65] Partnership with experts [56,61] Partnership with patients and their families [35] Linking researchers with community representatives [51] Public-private partnerships/ public engagement [39,43] *Diverse/Inclusive Stakeholder Engagement* Cultural awareness/engagement/diversity and community engagement [53] Need "an expanded network of partners that includes full representation from all regions, and possibly other disciplines" [37] Diverse representation and inclusion within collaborative platforms/networks [37,45,52,56] **Existing Resources** ***Human Resources/Skilled Professional****s* Resources available and accessible, including Human Resource allocation and existing relationships [35,44,45,54,77] Reposition of supplies to high risk areas [41] ***Financial Resources/Funding*** Access to regional and international investors [49] Third party coordinating supported public-private mixed projects with financial support [31,39] **Political environment** Political will to aid in the development/ institutionalization of effective collaborative structures [41,48,65] Political support for empowered decision making [72]	***Network Factors*** **Network Leadership** Support networks to identify a lead agency [41,52] Promote information sharing and joint decision-making across the network [49,60,65] Joint decision making, joint planning [60] Strong and engaged leadership [52] Multisectoral partners worked together for a common goal [47] Strategic risk communication with leadership [45] **Network Management** ***Task Management*** Task/ Case Management through MCMs [41] Convene regular multi-sectoral meetings [53,58,60] Shared response guidelines [52] Management protocol [58] Rapid startup response [42] Technical discussions held with community to support management systems [51] *Awareness* Awareness of systems in place, education/awareness, coordination, multidisciplinary info/data sharing [31,38,44,55,60,70] Increased engagement [31,45] Joint/coordinated public communications [60,70] Health threat communication includes early notification [49] Team/Internal communication includes data and information sharing [41,76] Public communication includes public awareness [54] Public release of risk analysis reports [77] Joint interviews with stakeholders [70] Finding common ground especially in regions of conflict to ensure health equity [49] Sharing perspectives [53] Behavior change communication [41] Effective information dissemination *Communication* **Characteristics:** frequent and honest [44,45] Timely; Consistent [45]; Reflexive/ flexible [59]; Iterative feedback [53]; Clear purpose [31,44,70]; Prioritized [riskbased] [45] Trust [49]; Interdisciplinary [31,53]; Contextualized [51]; Streamlined [54,70] **Methods:** Communication through MCMs—pre-meetings, data collection and sharing, forum for info sharing [48,58] ICS methods supported multisectoral communication/ effort [60] Regularly scheduled meetings/ Multidisciplinary meetings established/Follow-up meeting [43,48,53,58,60] Established clear lines of communication [31,43,51,77,78] Diversity of methods and platforms such as press briefs, websites, tv, newspaper, teleconferencing, listserv, available contact list, local/ regional/ cross-border meetings, periodic reporting [44,45,49,53,58,62,77,78] [38] *Ongoing Stakeholder Engagement* Engagement of diverse stakeholders to reflect community needs [53,75] Community engagement around prevention and control activities and biosecurity measures [51] Bottom-up approach with involvement of all levels/Champion/advocates [52,55] Action plans were agreed to with the community] needs ie. planning and implementation [51] Public, community, local authorities, govt agencies, NGOs, patients [45,49] Public health agencies/programs, travelers, global partners, federal and non-federal agencies [45] Civil-military; military/ foreign military involvement provided necessary support for other sectors [39,71,79] **Monitoring and Evaluation** Monitoring goals [35] Iterative review of collaborative processes [55,60] Monitoring and evaluation to show the outcome of interventions as beneficial or not [31,45,48] Research to understand outreach effectiveness [38] **Resource mobilization & allocation** ***Material distribution*** Established supply location [standardized, accessible, risk-based strategy); Subcommittee assigned to monitor supplies [41] Accessibility, standardized location, allocation, flow, product deployment [34,68,80] **Human Resource mobilization** Reflexive HR Protocol to ensure positions are adequately filled and that workers are incentivized [31,57] Additional military support allowed struggling organizations to leverage support and stay involved[71] Online recruitment [45]

The researchers also coded each paper for outcomes. Of all the articles coded, only 4 articles reported on outcomes of collaborations. The outcomes reported included: (1) cost reduction; (2) decreased mortality; (3) decreased morbidity, (4) multisectoral development opportunities resulted from the collaboration; (5) Improved safety; (6) effective use of available resources.

## Discussion

In this discussion, the research team suggests 12 thematic factors that may be used by practitioners involved in One Health activities to more systematically assess the successes and challenges of multisectoral collaboration, including those contributing to successful outcomes. Further research is needed to refine and validate these factors and ultimately support more uniform and rigorous assessments of One Health collaborations.

### Collaborative success factors categorized as starting conditions or process-based factors

The axial coding process allowed for factors reported to facilitate or discourage successful collaboration to be categorized as either a relevant *starting condition* of collaboration, or as relevant to the *process* of collaboration. During the data analysis, certain themes within each category of individual, organizational and network factors emerged as relevant to “setting the stage” for effective collaborative processes, while other factors were essential to maintaining the process of collaboration itself. The researchers found that this distinction was critical in our understanding of how successful collaborative processes are initiated. The starting conditions presented in this paper represent the collaborative preparedness and planning necessary to support effective One Health processes. In addition, the process of collaboration allows for the emergence of new ways of collaborating. This symbiotic relationship between starting conditions and process, allows us to view the entire system of collaboration. In this system, starting conditions influence the process of collaboration, and the process itself can lead to improvement of structures and processes that will now inform improved starting conditions. This cyclical and emergent process is inherent in collaboration and must be accounted for when considering evaluation and systems-based improvements.

#### Individual factors

Relevant success factors at the onset of a One Health event include an individual’s education and training, as well as prior experience and existing relationships. Many authors identified existing or previous education and training as enabling factors for collaborative success [37,40–42,44,47,49,51]. Formal technical education and training of individual workers prior to a health event was critical to prepare the necessary human resources for response efforts. Authors noted that foundational technical training during an event was often not possible [41,42], but that preemptive and collaborative planning did support the development of key relationships, and in some cases, the development of shared protocols used in the response. The absence of formalized training opportunities before an event, both individual technical training and collaborative, were frequently reported as a gap and a challenge to effective One Health response [40–42, 49]. Shared competencies were suggested as a strategy for standardizing protocols and performance across multiple individuals and organizations [49]. Multiple sources also reported the importance of prior experience in collaborative response efforts and how this established existing relationships to support the work, both formal (i.e. required communication through standard operating procedures) and informal (i.e. loosely structured and based on personal relationships and ongoing professional engagement) [34,45,52,53,56,57]. When instituted before a health event occurs, these starting conditions were reported to support a more effective collaboration processes.

Individual factors that supported the process of collaboration were most frequently reported as workers having access to necessary education and training that was available *ad hoc* or as “just in time” training to support operations during the health event. Examples reported include the use of shared training across organizations to additionally support institutional alignment and partnership with community-level organizations to provide training [39,42,49]. Many of these trainings were reported to be rigorous and responsive with continuous follow-up to support compliance [31,45,58]. Williams et al [45] discussed how ongoing multisectoral disease specific training supported workers to operate within new and existing systems while simultaneously sharpening their technical competence. These training and capacity-building opportunities were reported to provide a platform for better collaboration for outbreak response [49]. However, *ad hoc* trainings do not replace or diminish the need for foundational technical training, as formalized education and training were reported as a critical starting condition to facilitate quick mobilization in the case of a health event. Our literature review uncovered the role for both strong university-based education and training, and the role that *ad hoc* or “just-in-time” training can play to meet immediate operational needs during process-based response.

#### Organizational factors

Factors reported to enable organizational-level collaboration were broadly applicable to both the starting conditions and the processes of collaboration. Organizational structures, culture and resources were cited as important elements for creating an enabling environment for effective One Health collaboration. The organizations serve to connect the individual worker with a network of One Health actors.

The organizational structures that support collaboration were often discussed as a success factor. These structures include, but are not limited to, the policies and protocols or systems established within organizations to support technical implementation and collaborative efforts. Policies and protocols reported to be supportive included technical guidelines and standard operating procedures, as well as management, response and communication strategies and protocols [34,42,50,59,60,62]. In addition, organizations reported the need for functional systems for information and resource management and sharing and reporting both surveillance and laboratory results [41,43,45,48,55,59,63,64,66,67]. These systems were reported to benefit from being adaptive, flexible/reflexive and improved through iterative feedback and monitoring and evaluation [38,46,48,60].

Organizational culture was reported in multiple key areas [31,35,38,41,48,49,60,61,68–70,81]. The role of organizational leadership was discussed at length in many of the reviewed publications. Authors recognized and identified the importance of having strong and engaged leadership [31,34,52,68] and the need for leadership to support the iterative and developmental review of collaborative processes [60]. In addition, organizations benefited from having a culture that supported accountability, ownership, cultural engagement and diversity [53,68]. Trust and credibility were consistently reported as a key element of organizational success [38,41,49,70], as was the need for both an understanding of each other's processes and systems based thinking [34,48,53,70]. Authors reflected on the importance of cultural awareness, transparency of communication processes [31,34,53,61] and the engagement of diverse stakeholders who were able to reflect community needs [53].

Human resource-related factors appeared in all three levels of analysis. Research suggests that workers need to be trained at an individual level, have defined roles and responsibilities at an organizational level, and need to be able to mobilize their efforts at a network level. At an organizational level, Human resources are made up of individual contributors and also function as collective entities that reflect employees’ prior experiences, existing relationships, and the collective institutional knowledge of its members [31,34,45,49] serve to benefit the organizations in which they work. Clear roles and responsibilities were consistently reported [34,42,45], as well as awareness of systems in place to support ongoing engagement, operations and information sharing [31,45]. Additionally, several authors highlighted the importance of a reflexive workforce, i.e. human resources that were readily available and could be mobilized quickly and efficiently to respond to health event in a rapidly changing context [31,42,45,57].

#### Network factors

Starting condition factors reported to enable collaboration at the network level included network structures, existing relationships, available resources in the face of a health event, and the political environment in place to support these efforts. Pre-existing network structures were reported to provide a foundation for effective collaborative efforts to occur across participating organizations. Established Multisectoral Coordinating Mechanisms (MCMs), also referred to as One Health platforms or joint task forces, were often reported as key to assisting with collaboration across a network [34,41,42,45,48,52,60,72]. Organizational and network structures provided operational standards that crossed relational and organizational boundaries at all levels of the system—individual, organizational and network—which supported the development of formal relationships at each level. Analysis suggested that these systems and relationships need to be in place before the health event. MCMs provide a formalized operating foundation in which organizations and individuals could contribute, and formalized roles and responsibilities supported effective human resource mobilization in both organizations and networks [34,42,45,72]. These structures were often supported by formal policies or agreements such as bilateral agreements or Memoranda of Understanding (MOUs) [41,45,72]. In addition, operating procedures such as the Incident Command System (ICS) also supported effective mobilization of multiple organizations within the MCM [60]. Finally, the importance of formal structures were repeatedly emphasized as a response to “lessons learned” during challenging responses. On the contrary, lack of existing structures was reported to prevent efficient multisectoral engagement in the preparedness and response to health events [37,42,71]. Several sources indicated that reporting structures and policies at local, regional, national and international levels support continuity of response and effective implementation in response to health events [45,48,49,58,60,62,65,72,73]. These reporting structures and policies allowed for information flow between stakeholders, and the coordination of response efforts across a diversity of individuals and organizations participating in preparedness or response efforts [31,49,58,65,74].

Established structures created a foundation for network relationships that support effective outbreak response to a health event [31,40,42,45,47,49,54]. Development of formal and informal relationships prior to a health event allowed individuals, organizations and networks to more effectively respond once an emergency arose. The existence of structural agreements in any form such as MCMs, MOUs, shared Standard Operating Procedures (SOPs) or bilateral agreements were reported to support the further development of existing relationships to promote ongoing engagement prior to and throughout a health event [55,74]. Preemptive planning for potential disease threats was reported to strengthen connections and relationships and support multisectoral disease training, sometimes leading to shared protocols [45]. Additionally, the creation of common goals [34,47] and clearly defined, previously established, roles and responsibilities for individual actors and network partners were reported as necessary in network operations [34,40,42,45,49,54]. Cultural awareness and the inclusion of diverse stakeholders from government, nonprofit, and private sectors from the national to community level, was consistently reported as a success factor for collaborative efforts if included from the start [33,34,37,42,51,52,56,59,61,75].

Availability of resources, including human resources, that can be easily and efficiently mobilized in a health event was considered an important factor for response [31,34,37,41,44,45,49,52,54,74,82]. Authors also noted the importance of a supportive political environment to aid in the development and institutionalization of effective collaborative structures [41,48,65,72]. A supportive political environment was reported to enable the flow of available financial, human and material resources and empowered decision making [72]. Readily available resources supported rapid mobilization of collaborative efforts when a health emergency occurred. This is particularly impactful given that the absence of these resources and actions was noted across the literature as challenges to effective health response.

Network leadership and management processes were critical to effective multisectoral response efforts. Leadership engagement during a health event allowed for the mobilization and needed support for management processes. By utilizing existing structures and decision-making power, leaders and lead agencies can support managers and the process of management across organizations and networks. Emergency response protocols, such as the ICS, were frequently reported as mechanisms to this end, by concretely providing a leadership and management structure to support ongoing multi-organizational response. It was particularly useful for identifying a lead agency and establishing structures for regular meetings and communications. In the process of collaboration, relevant network factors included network leadership, management, and the effective and efficient mobilization of resources for response. For example, strong and engaged network leadership was noted as an important success factor for collaboration. When established prior to a health event, factors reported to support network collaborations included identifying a lead agency [41,52], promoting information sharing and joint decision-making across a network [49,60,65], and convening regular multisectoral meetings [53,58,60]. In addition, strong leadership was integral for strategic risk communication across the network [45].

Effective network management during an outbreak was reported in the areas of management practices, monitoring and evaluation (M&E), communication, awareness and ongoing stakeholder engagement. Management practices included case and task management through the MCM [41], regularly scheduled meetings [53,58,60] and development of shared response guidelines and management protocols across the network [58,81]. These management practices, when paired with existing structures, can support rapid start up response in the face of health events [51]. Monitoring and evaluation allowed for the iterative review of the collaborative processes during response efforts, as well as the outcomes of the collaborative process [31,34,38,45,48,55,60]. Monitoring and evaluation was reported as integral in being able to show the outcome of interventions as beneficial or not [31,38,45].

The importance of communications cannot be overemphasized and was repeatedly reported as an integral factor for building relationships, trust and supportive organizational culture, and for contributing to effective response processes. Both the characteristics and the methods of communications were highlighted as important. Characteristics of successful communication included the need to be frequent and honest [44,45]; timely and consistent [45]; reflexive and flexible [59] and prioritized (risk-based) [45], and streamlined [54,70]. Additionally, characteristics included the need for communications that build trust [49] and have a clear purpose [31,44,70]. These elements were widely reported to support effective communication within and across organizations [31,41,44,48,49,51,53,54,58,60,70,77,78,80].

Communication was deemed most effective when it was regular, frequent, and designed to foster awareness and support the engagement of a range of stakeholders, from local through national, regional and international levels. The MCMs, or the use of ICS, were often cited as important organizing structures for ongoing communication during a health event, supporting meetings, data collection and information sharing [43,48,58,60], underscoring the importance of starting conditions to support communications. Multiple methods of communication were reported including electronic communications, list-servs, contact lists and regular meetings; in many cases these were supported through existing MCMs [48,58] Monthly meetings [53,58,60] and establishing clear lines of communication [31,44,51,77,78] were reported as critical. These methods were supported by the use of a variety of methods and platforms such as press briefings, websites, television, newspaper, teleconferencing, listserv, available contact lists, local/ regional/ cross-border meetings and periodic reporting [44,45,49,53,58,62,77]. Additionally, leadership and management processes played a key role in supporting or challenging communication; high-level support, resource allocation, and use of good practices across an organization are foundational for good internal and external communication.

Closely linked with communication was the reported importance of building shared awareness and diverse stakeholder engagement. Awareness included information sharing, education campaigns, jointly coordinated communications and public release of reports with all members of the network and with the public [31,38,44,45,52,54,55,61,70]. Engagement of diverse stakeholders before, during, and after the response was reported as essential; these stakeholders included community and local actors, national governments, intergovernmental organizations, and operating partners [45,49,51–53,75]. To facilitate communication across stakeholder groups, Adams et al. [60] and Butler et al. [70] underscored the importance of transparent joint communications specifically between responders and community leaders for efficient and effective response. Butler et al. [70] further reported the success of joint interviews held with stakeholders to support shared understanding of response needs. Diverse partners, including foreign militaries, were reported to support foundational infrastructure that allowed other international partners to stay involved when supporting a response effort when they would not have been able to serve effectively on their own [39,71,79]. Common goals, common interests, and perspective sharing amongst stakeholders were reported to support an effective response to a health event [38,49,53].

Resource mobilization and allocation during an event, relies heavily on the starting conditions, as well as the communication, leadership and management during the process of collaboration. A number of authors pointed to the importance of being able to mobilize both the material and human resources. Once again, the involvement of diverse stakeholders, the use of MCMs and management systems such as ICS were attributed with the ability to draw upon existing resources. Processes characterized as successful included establishing a supply chain with standardized access, delivery, allocation and flow [34,41,68,80]. Human resource mobilization benefited from online recruitment [45] as well as reflexive Human Resource protocols to ensure that positions were filled and workers are incentivized and rewarded for participation [31,57].

#### Outcomes reported

Although the researchers created a code to capture reported outcomes of collaborative efforts, only a small number of authors reported outcomes of their collaborative processes. Outcomes were consistently missing or under-reported in the literature reviewed, and this is likely a result of One Health outcomes being difficult to characterize. The few reported included the outcomes of cost reduction and improved safety [34], decreased mortality [41], reduction in MRSA (Methicillin-resistant Staphylococcus aureus) cases [31], increased stakeholder buy-in [45], and a report that multisectoral professional development opportunities resulted from the response [47]. However, implementation of M&E activities was one of the major gaps in the reports of One Health collaboration. The majority of articles reviewed never discussed the evaluation of either the process of collaboration or the resulting outputs or outcomes. This creates a pivotal challenge in understanding how to improve One Health operations. The authors noted that outcomes of collaborative efforts were consistently missing or underreported in the literature reviewed.

#### Language in collaboration

Language used to describe One Health work continues to be a challenge when working across disciplines. Each discipline contributing, and specifically those authors reporting on these interactions, bring their own nomenclature and vernacular [36]. As also discussed in the limitations of this work, we encountered challenges in how authors reported on which entities were involved in the response to a health event. Organizations, sectors and disciplines were characterized in different ways, making is difficult to find a standard classifying system for the coding.

#### Considerations for the evaluation of One Health

Despite an emphasis on the importance of iterative improvements to collaboration, the implementation of monitoring and evaluation activities was one of the major gaps in the reports of One Health collaboration. The majority of articles reviewed never discussed the evaluation of either the process of collaboration or the resulting outputs or outcomes. This creates a pivotal challenge in understanding how to improve One Health operations. It became clear in the literature review that there was no standard framework for how to evaluate One Health processes [12, 36]. Although networks and collaborators such as the Network on the Evaluation of One Health are making important advances, practical evaluation tools are still needed [83]. Some authors from public affairs, such as Emerson and Nabatchi (2015) et al.[19] have proposed a framework for evaluating outputs, outcomes, and what they refer to as “adaptations” of collaborative processes [18,19]. Their work is one of the first to propose an integrated framework that captures collaborative results at all levels of the system, from the target population to the participating organizations, and the network as a whole. The results of this scoping review are intended to support the next steps in supporting One Health evaluation.

### A proposed framework for analyzing and reporting on One Health collaborations

Using the 12 factors uncovered in this review, the authors have outlined a reporting framework (Table 9) that may help practitioners consider their activities in light of important collaborative starting conditions and process-based factors. The researchers propose this to the One Health community as a tangible next step that may lead to more effective reporting and potentially evaluation of One Health efforts in the future. The proposed framework in Table 9 recognizes that each factor will be operationalized within the context of the health event and that flexibility in reporting is imperative. This framework may be useful in providing a common language on how practitioners discuss and report on their One Health efforts.

**Table 9:** A proposed reporting framework based on the 12 identified factors.

**Multisectoral Event/ Activity**:_________________________________________________________ Organizations that participated: Disciplines represented: *Discipline is defined as a branch of knowledge (e.g. economics, virology, epidemiology, law, clinical medicine, vector biology). Objectives of the collaboration (if available):		
Starting Conditions: Describe the following factors as they existed **prior** to the health event/activity: • **Human resources available** Disciplines and levels of technical or collaborative training in place • **Structures in place** network and/or organizational structures (ie. MCMs, MOUs, Policies, Protocols, technical systems, etc.) • **Existing relationships** formal and informal as they exist across individuals, organizations or networks • **Resources available** resources readily available and accessible • **Political Environment** how this influenced the One Health system at work • Other:_______________________________	Successes	Challenges
Process-based factors: Describe the following factors as they existed **during** the health event/activity: • **Training available** training available and utilized to support the process • **Leadership processes** leadership at the organizational and network level. How did the leaders support effective One Health responses • **Management processes** management processes during the health event • **Communication processes** methods used and engagement of stakeholders • **Resource mobilization** How resources were mobilized? • **Monitoring and evaluation** how were collaborations were evaluated? • Other:____________________________	Successes	Challenges
Output indicators: *What are the measurable effects of the collaborative effort*? *(eg*. *number of schistosomiasis educational fliers distributed to community healthcare workers)* Outcome indicators: *What changes are expected as a result of the One Health effort*? *(eg*. *reduced incidence of schistosomiasis at the community-level 6 months post campaign)* Suggested improvements for future collaborations:		

### Lessons in collaboration from a transdisciplinary research team

In the process of conducting this research, the research team encountered many of that same collaborative challenges as described in the articles reviewed. The research team had to negotiate and re-negotiate ways of working, integrate differing points of view and assign roles in ways to leverage expertise but not reinforce bias. Additionally, the researchers had to establish and meet internal standards while also achieving the outward facing objective of finishing the analysis and writing of this article. Finally, as with any transdisciplinary work, language was consistently a problem. The inherent challenge of interdisciplinary work is in how we talk about collaboration and the terminology we use to describe both theory and practice. For the research team, creating clear definitions supported the development of a common language.

Differing approaches can be a significant barrier when active collaboration is not structurally supported, valued, and continuously monitored for health and effectiveness. Our efforts reinforce the need for training for those skill sets that fall beyond technical sector-specific training. When grappling with the question of which skills were most important in our collaborative process, we determined that the shared objective of collaboration was the foundation for our ability to integrate the differing expertise that each team member brought to the process. Simply, we took continual action to achieve our combined goal including reading new literature, considering new frameworks, learning new things, and asking many questions. The subsequent challenge is, of course, that there are very few formal opportunities to gain access to training around these competencies and mindsets in One Health teams. Most often, as in our case, it is an *ad hoc* process that rests on the motivations, shared values, and time available within a team to develop in this way. Our review suggests that, while this approach worked for us, it would not be a time or resource-effective approach within the context of a health emergency. Thus, One Health approaches need to be evaluated to help practitioners decide when and how to most effectively collaborate for their intended purposes.

## Conclusions

Of the 2,630 article abstracts screened, only 179 met initial inclusion criteria and the full research articles were obtained. Of that subset of articles, only 50 discussed the successes, challenges and lessons learned from operational One Health response to a health event. A majority of the articles focused broadly on the need for collaboration between multiple sectors or disciplines with little attention to what factors enable an effective One Health response effort. The low number of included articles reflects a broader challenge for the One Health community, suggesting the necessity that One Health researchers move beyond discussing the inherent need for One Health, to actually reporting on the processes, outputs and outcomes of their collaborative efforts. As such, no consistent framework or language was found to report on the process, outputs or the outcomes of One Health work in the articles reviewed. In the analysis, the research uncovered 12 factors that supported successful health event response. The researchers were able to make important advances by characterizing these factors as important at the start of collaboration or relevant to the process of collaboration. Using these 12 factors, the researchers propose a One Health reporting framework which when used to report on One Health collaborations, can support the further refinement and identification of success factors for One Health. These factors may serve as the basis for developing evaluation metrics and the iterative improvement of One Health processes around the globe.

This publication is made possible by the generous support of the American people through the United States Agency for International Development (USAID). The contents are the responsibility of the authors and do not necessarily reflect the views of USAID or the United States Government.

## Supporting information

S1 File PRISMA 2009 checklist. (DOC)
